# Accurate, strong, and stable reporting of choroid plexus epithelial cells in transgenic mice using a human transthyretin BAC

**DOI:** 10.1186/s12987-018-0107-4

**Published:** 2018-08-16

**Authors:** Brett A. Johnson, Margaret Coutts, Hillary M. Vo, Xinya Hao, Nida Fatima, Maria J. Rivera, Robert J. Sims, Michael J. Neel, Young-Jin Kang, Edwin S. Monuki

**Affiliations:** 1 Department of Pathology and Laboratory Medicine, UC Irvine, Irvine, USA; 2 Sue and Bill Gross Stem Cell Research Center, UC Irvine, Irvine, USA; 3 Department of Developmental and Cell Biology, UC Irvine, Irvine, USA; 4 Department of Biological Sciences, California State University, Long Beach, USA

**Keywords:** Transthyretin, Choroid plexus, Cerebrospinal fluid, Retinal pigment epithelium, Hepatocytes, Islets

## Abstract

**Background:**

Choroid plexus epithelial cells express high levels of transthyretin, produce cerebrospinal fluid and many of its proteins, and make up the blood-cerebrospinal fluid barrier. Choroid plexus epithelial cells are vital to brain health and may be involved in neurological diseases. Transgenic mice containing fluorescent and luminescent reporters of these cells would facilitate their study in health and disease, but prior transgenic reporters lost expression over the early postnatal period.

**Methods:**

Human bacterial artificial chromosomes in which the transthyretin coding sequence was replaced with DNA for tdTomato or luciferase 2 were used in pronuclear injections to produce transgenic mice. These mice were characterized by visualizing red fluorescence, immunostaining, real-time reverse transcription polymerase chain reaction, and luciferase enzyme assay.

**Results:**

Reporters were faithfully expressed in cells that express transthyretin constitutively, including choroid plexus epithelial cells, retinal pigment epithelium, pancreatic islets, and liver. Expression of tdTomato in choroid plexus began at the appropriate embryonic age, being detectable by E11.5. Relative levels of tdTomato transcript in the liver and choroid plexus paralleled relative levels of transcripts for transthyretin. Expression remained robust over the first postnatal year, although choroid plexus transcripts of tdTomato declined slightly with age whereas transthyretin remained constant. TdTomato expression patterns were consistent across three founder lines, displayed no sex differences, and were stable across several generations. Two of the tdTomato lines were bred to homozygosity, and homozygous mice are healthy and fertile. The usefulness of tdTomato reporters in visualizing and analyzing live Transwell cultures was demonstrated. Luciferase activity was very high in homogenates of choroid plexus and continued to be expressed through adulthood. Luciferase also was detectable in eye and pancreas.

**Conclusions:**

Transgenic mice bearing fluorescent and luminescent reporters of transthyretin should prove useful for tracking transplanted choroid plexus epithelial cells, for purifying the cells, and for reporting their derivation from stem cells. They also should prove useful for studying transthyretin synthesis by other cell types, as transthyretin has been implicated in many functions and conditions, including clearance of β-amyloid peptides associated with Alzheimer’s disease, heat shock in neurons, processing of neuropeptides, nerve regeneration, astrocyte metabolism, and transthyretin amyloidosis.

## Background

Choroid plexus epithelial cells (CPECS) represent an under-studied cell type that contributes vitally to the health of the brain. CPECs, which reside in all four brain ventricles, produce cerebrospinal fluid (CSF), pumping water from the blood into the ventricles and manufacturing and secreting a variety of important CSF proteins such as growth factors and the thyroxine-carrier transthyretin (TTR) [1, 2]. The CSF in turn mechanically cushions the brain and provides a circulatory system communicating with brain interstitial fluid, both delivering beneficial substances and removing metabolic waste [2, 3]. Tight junctions between the CPECs are responsible for establishing the blood-CSF barrier [4], and yet CPECs also possess various transport systems that actively insure the delivery of certain substances from the blood into the CPECs as well as into the CSF [5, 6].

Choroid plexus epithelial cells decline in number and health with age and in association with several neurological diseases [1, 7, 8]. Indeed, deterioration of CPECs has been hypothesized to contribute to the progression of these diseases, with a particular emphasis on the potential role of TTR in the removal of β-amyloid in Alzheimer’s disease [7–9]. CPECs may also be critically involved in immune cell activation and entry into the CNS in the development of multiple sclerosis and other inflammatory conditions [10, 11]. On the other hand, the discovery both that CPECs can be derived from human stem cells and that they can become integrated into the recipient choroid plexus (ChP) after transplantation suggests a potential use of engineered CPECs in cell-based therapies for neurological disorders [12].

To better explore the basic biology of CPECs, track them in disease models, monitor their differentiation from stem cells, purify them, and assess their integration, survival, and health after transplantation, it would be useful to have transgenic mice in which these cells express fluorescent and/or luminescent reporters. The most abundant and selective mRNA in the CPEC transcriptome is TTR [13, 14], which is produced in a limited set of other tissues including the liver, pancreas, and retinal pigment epithelium [15], as well as in neurons as a heat shock protein under conditions of stress and disease [16]. TTR’s abundance in CPECs, its tissue selectivity, and its long-term expression suggest that its gene regulatory sequences would be effective for directing the expression of CPEC reporter proteins.

To study the development of the visceral endoderm, Kwon and Hadjantonakis previously created transgenic mice using a plasmid vector in which a red fluorescent protein (mRFP1) was expressed under the direction of 3 kb upstream regulatory sequence of the mouse TTR gene [17]. Expression of RFP in these mice did occur in CPECs of embryonic and neonatal mice and were useful for monitoring initial CPEC derivation from stem cells [12] and fluorescence activated cell sorting of CPECs from young mice [14]; however, the expression in embryonic choroid plexus was found to be mosaic and to decline markedly with age [14].

In the present paper, we describe the generation of transgenic CPEC reporter mice using a human bacterial artificial chromosome (BAC). The large expanse of DNA in a BAC transgene not only holds the promise of including more extensive TTR regulatory sequences, but also should preclude the type of positional effects that plague the use of smaller transgenic constructs, such as ectopic and mosaic expression as well as extinction [18]. We characterize these mice with special attention to CPECs to establish that the reporters accurately reflect the tissue distribution of TTR, that they display an appropriate embryonic developmental profile, and that they continue to be expressed well into adulthood. We conclude that these mice will be useful tools for the investigation of not only CPECs but also other cell types expressing TTR.

## Methods

### Recombineering of a human TTR BAC

The RP11-571I2 BAC, which contains the human TTR gene and flanking 5′- and 3′-proximal genomic sequences of 126 and 41 kb, respectively, was obtained from CHORI BAC PAC Resources Center (https://bacpacresources.org/) and recombineered by the UC Davis Mouse Biology Program to replace the coding sequence of the human TTR gene (from start codon to stop codon, including introns) with either tdTomato or luciferase2 (luc2) cDNA. The clones were constructed using BAC recombineering methods adapted from Chan et al. [19], using the pSIM18 plasmid and Wang et al. [20], using the RspL counter selection method.

The TTR coding sequence in RP11-571I2 was first replaced with the RspL counter selection cassette in opposite orientation from TTR. (Previous attempts in the same orientation failed to yield stable intermediate clones.) *E. coli* clones in 96 well plates were PCR screened using primers spanning recombination junctions, with twelve subsequently verified by pulse field gel electrophoresis (PFGE) of SbfI digested BAC DNA. Two were then electroporated into *E. coli* DH10 cells and grown with kanamycin and chloramphenicol to separate the hTTR-RspKan-RC clone from unmodified BAC DNA, with all clones tested (four each for the two separation transformations) showing the expected SbfI restriction pattern by PFGE. To replace the RspL counter selection cassette with luc2 or tdTomato, the selected intermediate clone was made “recombineering-ready” by transformation with the pSIM18 plasmid, then luc2 or tdTomato cassettes were introduced and grown in chloramphenicol and streptomycin.

For luc2, PCR screening identified 14 putative clones, then pooled BAC DNA from these clones were transformed into DH10 cells to separate hTTR-luc2 and intermediate clones. Seven clones were selected based on PCR screening, and all seven were verified to be hTTR-luc2 clones by PFGE of SalI-digested DNA. The luc2 insert was also PCR amplified from all seven clones, and five were verified by sequencing for luc2 coding sequence integrity and TTR replacement. For tdTomato, BAC DNA from 6 PCR-verified clones were pooled for separation transformation into DH10 cells, with six hTTR-tdTomato clones verified by PFGE of SalI-digested BAC DNA and sequencing of tdTomato inserts. Subsequent restriction analysis of the tdTomato BAC by the UC Irvine Transgenic Mouse Facility indicated loss of an expected Sbf1 restriction site, which ultimately did not impact the generation of useful transgenic reporter lines.

### Generation of transgenic mice

Transgenic mice were produced by the UC Irvine Transgenic Mouse Facility by pronuclear injection of intact circular (tdTomato) or Sbf1-linearized (luc2) DNA. B6SJLF1/J mice were used as egg donors. Subsequent breeding used CD1 mice. Successful BAC insertion was judged by PCR of F1 genomic DNA employing primer pairs distributed throughout the BAC (Fig. 2, Table 1). Results reported here involve progeny of the original founders through four generations of CD1 breeding. Routine genotyping involved PCR of reporter coding sequences (Table 1). Both male and female mice were studied. Euthanasia involved either CO_2_ inhalation or intraperitoneal injection of Euthasol (Virbac AH, Inc., Fort Worth, TX).

**Table 1:** PCR primers used in the study

Category	Primer pair	Direction	Sequence (5′ to 3′)	Amplicon (bp)
genotyping	tdTom internal	Forward	CACCATCGTGGAACAGTACG	142
		Reverse	GCGCATGAACTCTTTGATGA	
	BAC 100 kb 5′ #1	Forward	TTAGGATTCAGGTGGCCTTG	148
		Reverse	TGCACATCCTTGGCAATAAA	
	BAC 100 kb 5′ #2	Forward	AATGAAGAGGCTGCCAAAGA	192
		Reverse	AGTGGATCCCACGACAGTTC	
	BAC 60 kb 5′	Forward	AAGCCCAAGATCAAAGCAGA	202
		Reverse	CTCACGTGCTGAAATCCTGA	
	BAC 40 kb 3′	Forward	TCAGCAGCTTCCTGCTACAC	500
		Reverse	GCTAGACAGGTACCCAGGGA	
	luc2 internal	Forward	ACAGAAACAACCAGCGCCATTCTG	590
		Reverse	TCCAACTTGCCGGTCAGTCCTTTA	
	JAX control	Forward	CTAGGCCACAGAATTGAAAGATCT	324
		Reverse	GTAGGTGGAAATTCTAGCATCATCC	
RT-qPCR	tdTomato	Forward	ATCGTGGAACAGTACGAGCG	133
		Reverse	TGAACTCTTTGATGACGGCCA	
	transthyretin	Forward	ATCGTACTGGAAGACACTTGGC	131
		Reverse	CCGTGGTGCTGTAGGAGTAT	
	18S ribosomal	Forward	CGGCTACCACATCCAAGGAA	187
		Reverse	GCTGGAATTACCGCGGCT	
	cyclophilin A	Forward	GAGCTGTTTGCAGACAAAGTTC	125
		Reverse	CCCTGGCACATGAATCCTGG	

### Analysis of mRNA levels by real-time reverse transcription quantitative PCR (RT-qPCR)

Choroid plexus was rapidly collected from lateral and fourth ventricles while brains were submerged in cold PBS, frozen together on the walls of microcentrifuge tubes sitting on dry ice, then stored at − 80 °C or lysed with 50–100 µL of HLY lysis buffer (Biomiga, San Diego, CA) added to tubes on dry ice followed by homogenization using plastic pestles. Additional HLY was added to a final volume of 350 µL, with further homogenization, and homogenates were stored at − 80 °C. Pieces of liver (20–40 mg) were also rapidly dissected and minced immediately after collection, followed by homogenization in 200 µL of cold HLY buffer. Additional HLY buffer was added to a final volume of 500–700 µL, with further homogenization, and homogenates were stored at − 80 °C.

Total RNA was isolated from homogenates of liver or ChP using Biomiga EZgene tissue RNA kits. Yield and purity were judged by absorbance at 230, 260 and 280 nm (NanoDrop 2000c spectrophotometer, ThermoFisher, Waltham, MA). cDNA was synthesized using random hexamers, dNTPs, RNAsin (Promega, Madison, WI), and Moloney Murine Leukemia Virus reverse transcriptase (Promega). For every sample, minus-RT control incubations were performed using DEPC-treated water in place of reverse transcriptase. RT-qPCR reactions used iTaq Universal SYBR Green Supermix (Bio-Rad, Hercules, CA) on a Roche LightCycler 480II in 96-well plate format, and primer pairs for tdTomato, TTR, cyclophilin A, or 18S ribosomal RNA (see Table 1) on samples and water-only controls. Primers were validated for log-linearity of Ct values using serially-diluted ChP and liver RNA samples, > 90% doubling efficiencies, homogeneous melt curves, and single amplicons of appropriate size by gel electrophoresis. Results were excluded if amplification was detected in water-only controls or if +RT and − RT samples displayed a Ct value difference of less than 10 cycles with the 18S primer set.

RT-qPCR results were reported as ΔΔCt, subtracting target Ct values (tdTomato and TTR) from the average of two reference Ct values (18S and cyclophilin A) for the same experimental sample [21], and then subtracting experimental sample values from the average value obtained across all samples of wild type mice (22 total). More positive values indicate more mRNA.

### Immunostaining and imaging

Brains or dissected tissues were fixed by submersion in 4% paraformaldehyde in phosphate-buffered saline (PBS) (4–48 h), followed by overnight cryoprotection in PBS containing 30% sucrose, both at 4 °C. Tissues then were embedded in O.C.T. compound (TissueTek, Sakura Finetek, Torrance, CA) in specimen molds, frozen on dry ice, and stored at − 80 °C until sectioning. Tissue blocks were sectioned at 20-µm thickness in a Leica CM3050 S cryostat, and slices were collected on Superfrost plus slides.

Sections were washed thrice with PBS (5 min/wash at room temperature), followed by blocking with 5% serum in PBS containing 0.3% Triton X-100 and overnight incubation in primary antibody at 4 °C. After three additional washes, incubations with secondary antibodies (1:500) were carried out for 1 h at room temperature, followed by three additional washes and mounting with Fluoromount-G containing DAPI fluorescent counterstain (SouthernBiotech, Birmingham, AL). Antibodies were diluted in 1% serum in PBS containing 0.3% Triton X-100. Primary antibodies were rabbit anti-RFP (1:1000, 600-401-379, Rockland, Limerick, PA), goat anti-anion exchange protein 2 (AE2, 1:250, sc46710, Santa Cruz Biotechnology, Dallas, TX), rabbit anti-glucagon (1:200, 2760S, Cell Signaling Technology, Danvers, MA), sheep anti-TTR (1:500, Abcam, ab9015), and guinea pig anti-insulin (1:500, Dako A0564, Agilent Technologies, Santa Clara, CA). Secondary antibodies (1:500, Life Technologies, ThermoFisher) included Alexa 488-conjugated donkey anti-rabbit (to detect anti-glucagon antibodies), donkey anti-goat, donkey anti-sheep, and goat anti-guinea pig IgG, as well as Alexa 555-conjugated donkey anti-rabbit IgG (to detect tdTomato).

Fluorescent (RFP filters) and brightfield imaging of fresh tissues used either an AMG EVOS fl microscope or a Nikon SMZ1500 dissecting fluorescent microscope and an SD-Ri1 digital camera. Immunofluorescence on slides was evaluated using a Nikon Eclipse E400 microscope fitted with DAPI, FITC, and TRITC filters and imaged using a Nuance FX camera (Perkin Elmer). Image contrasts were adjusted automatically by the Nuance software using the “Clip/Stretch” display function. Channels were blended in Adobe Photoshop, and further adjustments to contrast were made in Photoshop to give a more balanced representation of the channels. For comparative panels in figures, image adjustments were made in parallel.

### Primary transwell cultures of CPECs

Choroid plexus was rapidly dissected from the lateral and fourth ventricles of four P2 mice and collected in 500 µL ice-cold HBSS containing 0.6% sucrose. Enzymatic dissociation of CPECs was performed with 500 µL of 1000 U/mL collagenase II (Sigma 234155) in 3 mM CaCl_2_. The mixture was incubated at 37 °C for 40 min, with vigorous agitation every 5 min, at the end of which no large aggregates of tissue could be visualized by eye. After trituration, the suspension was then centrifuged at 805*g* for 3 min, the supernatant was discarded, and the pellet containing cells and small cell aggregates was resuspended in 500 µL TrypLE Express (Gibco 12605028) and incubated at 37 °C for 5 min. After trituration, this suspension was centrifuged at 805*g* for 3 min, the supernatant was discarded, the cell pellet was resuspended in 510 µL of CPEC media (DMEM/F12 (Gibco 11320033), 10% fetal bovine serum (Hyclone SH 300703), antimicrobial-antimycotic supplement (“anti-anti”, Gibco 15240062), and the cell suspension was plated in 0.33 cm^2^ Transwell chambers (Corning 3470) previously coated with poly-d-lysine and laminin and cultured for various durations, with media replacements every other day. Nutrient media was supplemented with 20 µM cytosine β-d arabinofuranoside (AraC, Sigma C6645) during days 0–4 of culture, and serum was withdrawn on day 4.

The EVOM2 apparatus equipped with the Endohm 6 attachment, specifically designed for use with 0.33 cm^2^ transwell inserts (World Precision Instruments) was used to measure trans-epithelial electrical resistance. Values of > 65 Ω cm^2^ have been reported as indicative of monolayer confluency for primary mouse CPEC culture [22]. Fluorescence (RFP filter) and phase contrast images were obtained using an AMG EVOS fl microscope.

### Luciferase assays

Lateral and fourth ventricle ChP from each single mouse were combined and homogenized in 100 µL of passive lysis buffer (PLB, Promega), using a plastic pestle in a microcentrifuge tube. Other tissues were homogenized in 1–2 mL of PLB using a PowerGen 125 tissue homogenizer (Fisher Scientific) typically set at medium speed, twice for 15 s each. Harder tissues such as heart, kidney, and eye were minced with scissors prior to homogenization. Homogenates were frozen at − 80 °C. Upon thawing, homogenates were centrifuged for 10 min at 10,000*g*, 4 °C, and the supernatants were taken for determination of protein concentration and luciferase activity. Protein was assayed using BioRad Bradford dye reagent and bovine serum albumin standards. Luciferase activity was measured using an E1500 assay kit (Promega). A BioTek Synergy HT microplate reader was used to measure absorbance in protein assays and luminescence in luciferase assays. Results were expressed as plate reader light units per mg of protein. The luciferase assay was determined to be linear across six tenfold serial dilutions of a sample yielding the maximum detected activity level.

## Results

### Loss of fluorescence in postnatal Ttr::RFP mice

**Fig. 1:**
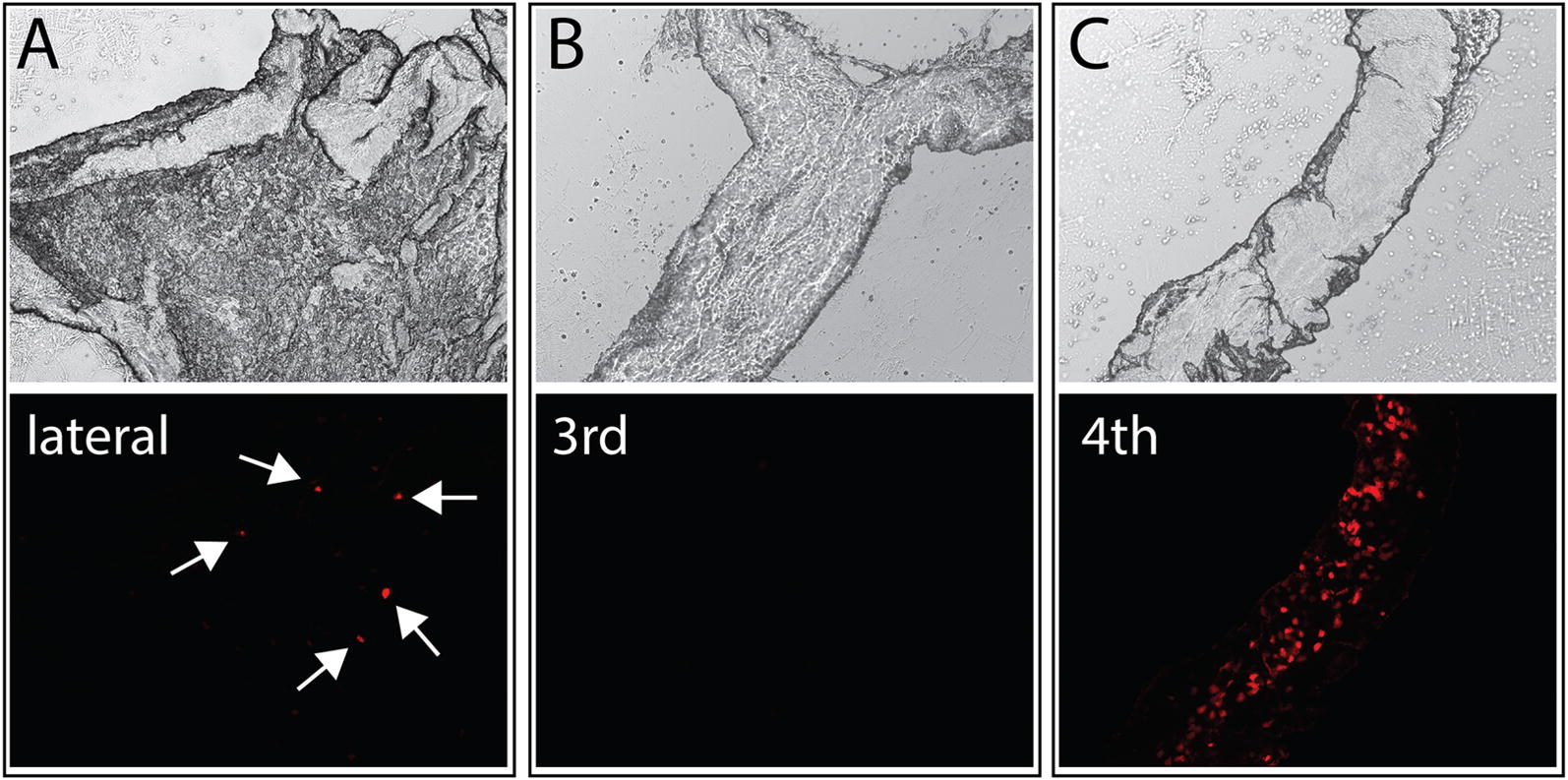
Choroid plexus fluorescence is diminished in weanling Ttr::RFP transgenic mice. Upper panels show images of transmitted light and bottom panels show corresponding images of red fluorescence taken for choroid plexus dissected from a 21-day old Ttr::RFP transgenic mouse. A few scattered fluorescent CPECs were visible in the lateral ventricle ChP (**A**, arrows), but none were found in the third ventricle ChP (**B**). A larger number of fluorescent CPECs were detected in the fourth ventricle ChP, although expression was clearly mosaic (**C**)

Although we confirmed the robust fluorescence of CPECs in embryonic and neonatal Ttr::RFP transgenic mice produced by integration of a plasmid vector [17], we found that RFP expression was mosaic in embryonic mouse ChP [14], and the number of cells expressing fluorescence declined precipitously after birth. Figure 1 shows ChP from a 3-week old Ttr::RFP mouse. Only a few sporadic, brightly labeled cells were still detectable in the lateral ventricle ChP (arrows) at this age, and the third ventricle ChP was devoid of fluorescence. Fewer than half of the CPECs in the fourth ventricle ChP were fluorescent. The loss of fluorescence indicated that the reporter was not tightly linked to TTR expression over the lifetime of the mice, making it incompatible with many of our intended uses. Thus, we were motivated to generate transgenic mice with more stable reporter expression that better matched TTR.

### Generation of human TTR BAC-tdTomato mice

**Fig. 2:**
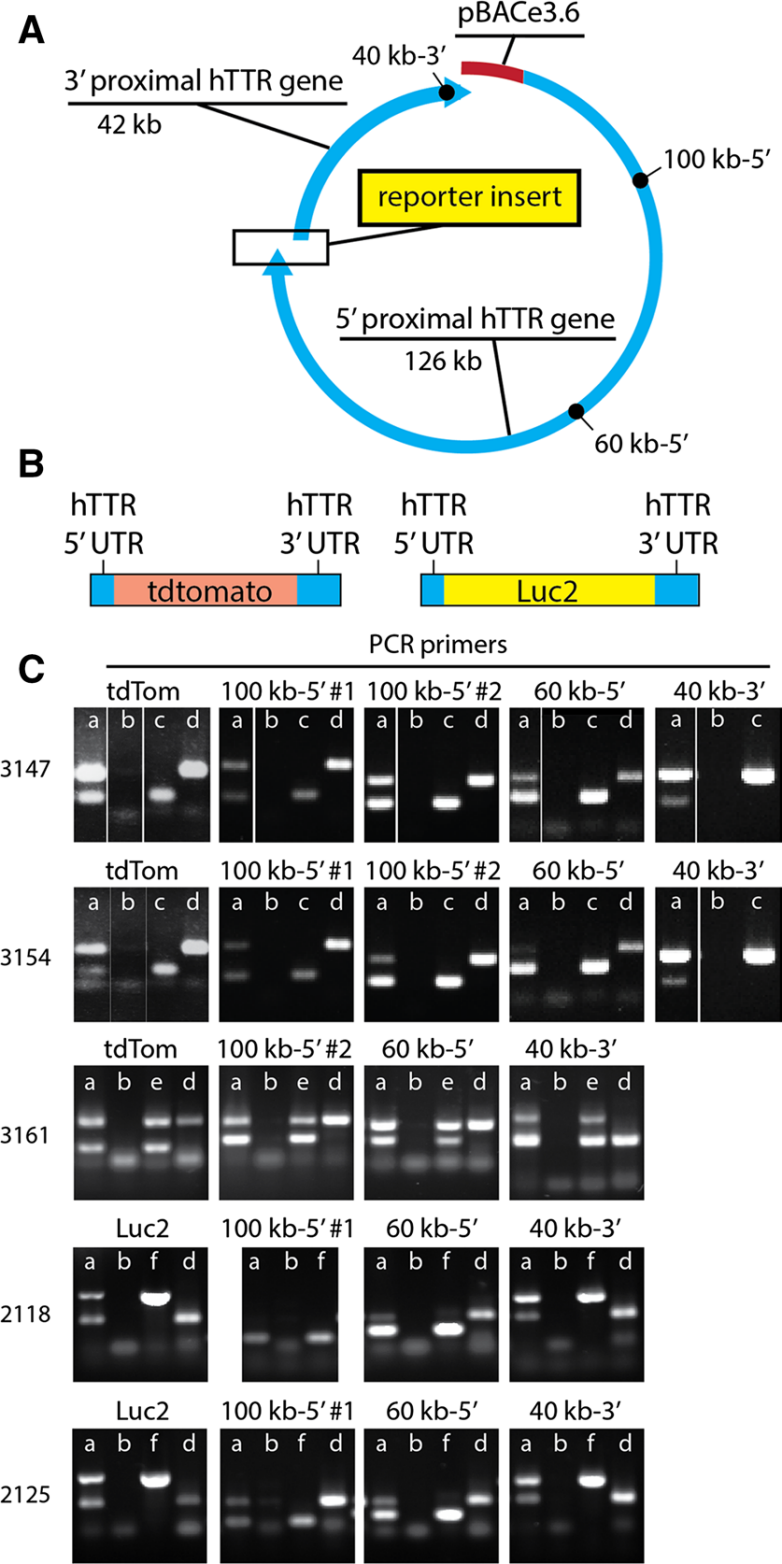
BAC constructs used to generate transgenic reporter mice. The RP11-571I2 BAC contains the human transthyretin (TTR) coding sequence as well as 42 kb of additional DNA in the 3′-direction and 126 kb of DNA in the 5′-direction (**A**). The TTR coding sequence from the start codon to stop codon, including introns, was replaced with reporter cDNA, but the 3′- and 5′-untranslated regions of the TTR mRNA remain (**B**). Sequences distributed throughout the reporter BAC are present in genomic DNA of transgenic mice (**C**). Founder lines are shown on the left. Sequences of primers are given in Table 1, and their locations within the BAC are designated by black circles in **A**. Each PCR reaction involved the use of two primer pairs, one amplifying a control sequence present in all mice and one amplifying sequences either within the reporter cDNA or representing human sequences in the BAC that are far removed from the insert in either the 5′- or 3′-direction. Amplicons were separated by electrophoresis in 1% agarose gels containing GreenGlo fluorescent DNA dye. Lanes labeled “a” represent reactions involving experimental transgenic mouse DNA; lanes “b” are negative controls in which water was substituted for DNA; lanes “c” are positive controls involving intact tdTomato BAC; lanes “d” are negative controls involving DNA from a wild type mouse; lanes “e” are positive controls involving DNA from a different, previously genotyped transgenic mouse; and lanes “f” are positive controls involving linearized luc2 BAC DNA

To confer more faithful regulation of expression, we decided to use as a starting point bacterial artificial chromosome (BAC) RP11-571I2, which contains the human TTR gene (Fig. 2A). A human BAC was chosen over mouse to enable interpretations of expression studies that more likely extend to human TTR, a protective and clinically relevant protein [23–26]. The contiguous TTR coding region (start codon to stop codon, including introns) was replaced with cDNA for either tdTomato or luc2, and transgenic mice were generated via random BAC integration following pronuclear injection into fertilized eggs (Fig. 2B; see “Methods” for details). 5′ and 3′ untranslated TTR regions, which contain likely transcriptional and mRNA stability elements, were retained. Tdtomato was chosen for its bright, red-shifted fluorescence [27], and luc2 was chosen to confer the possibility of repeated, highly sensitive in vivo imaging (and highly quantitative in vitro measurement) of emitted light in the presence of luciferin substrate and ATP [28].

A total of 63 mice were born from eggs injected with the tdTomato reporter BAC. Of these, 25 possessed tdTomato reporter sequences by PCR, and 21 were also PCR-positive for human TTR regulatory elements (100 kb upstream, 60 kb upstream, and 40 kb downstream of coding sequence; Fig. 2A, C, Table 1). Nine founder mice were then bred. All nine displayed germline transmission and showed red fluorescence in dissected, unfixed ChP (evaluated at P21–28). Of these, two founder lines yielded offspring with only weakly fluorescent ChP (RFP fluorescence barely detectable using an EVOS microscope at 10X, 100% lamp intensity, 1 s exposure), two yielded moderately fluorescent ChP (fluorescence clear at 10X, but not at 4X), and five yielded strongly fluorescent ChP (fluorescence clear at 4X). Three of these strongly fluorescent founder lines were bred further, and hemizygous mice from these lines were characterized in greater detail in this paper (founder lines 3147, 3154, and 3161). All three lines have been archived by cryopreservation of sperm that have been verified for fertility after thawing.

**Fig. 3:**
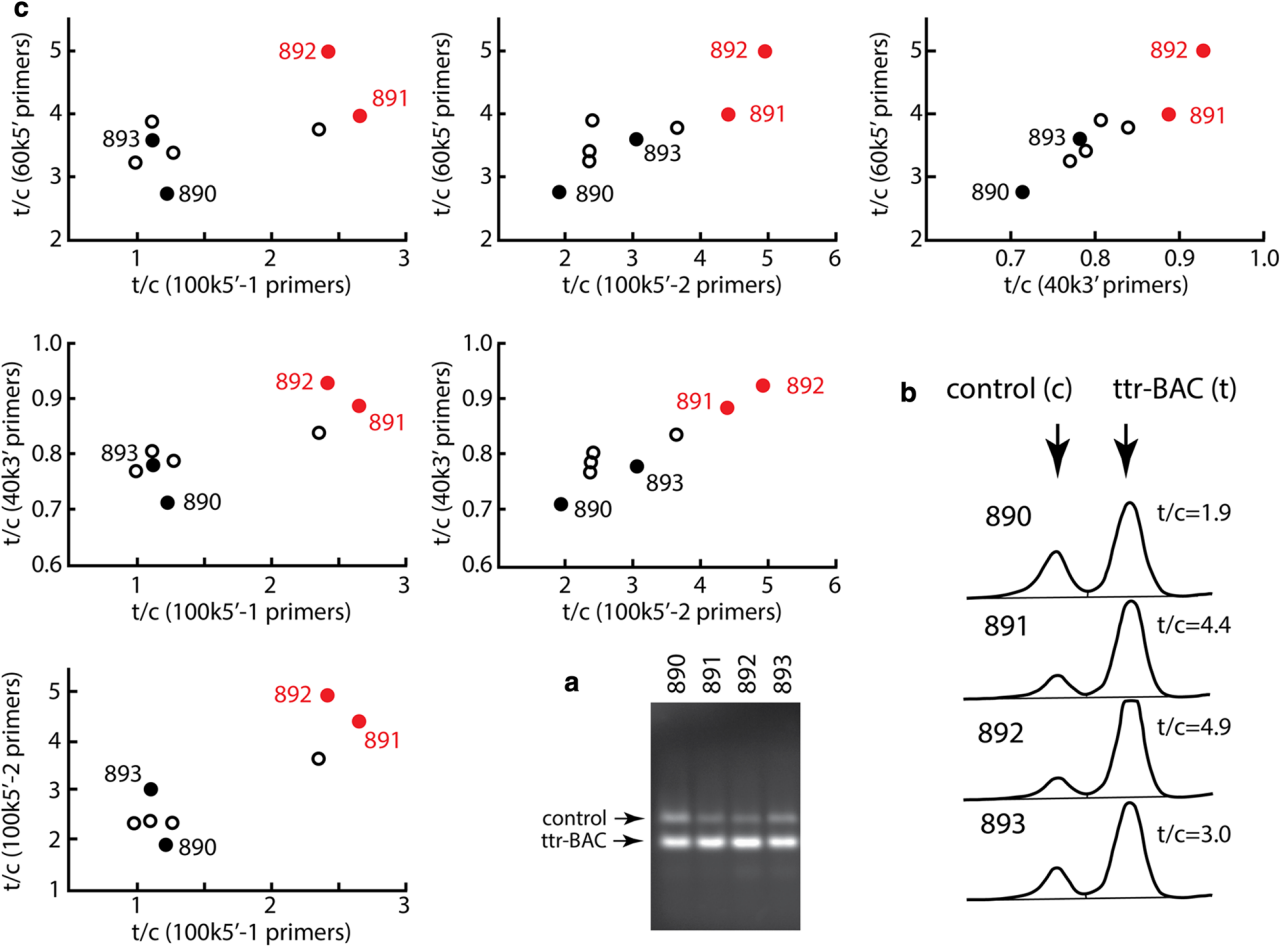
Homozygous mice were identified using traditional PCR of genomic DNA. Oligonucleotide primers designed to amplify parts of the TTR-tdTomato BAC were combined in PCR reactions with primers to amplify control sequences present in all mice. These reactions were subjected to conventional 1% agarose gel electrophoresis (**a**). Images of the gels were then analyzed as densitometry scans using Image J, and the relative areas under the ttr-BAC amplicon band (t) and the control amplicon band (c) were calculated as a ratio (**b**). The ratios obtained for any one BAC sequence were plotted against the ratios for each other BAC sequence (**c**). Certain mice from a given litter tended to have high ratios of BAC to control amplicons across all primer pairs, yielding distinct clusters in these plots (e.g., mice 891 and 892 in **c**). Nine out of ten mice identified this way, including 891 and 892, were verified to be homozygous by breeding with wild-type mates. The differences in ratios typically reflected both an increase in the fluorescence intensity of the BAC amplicon and a decrease in intensity of the control amplicon for the homozygous mice (**b**), suggesting competition between these reactions

Pairs of hemizygous mice from each of the three founder lines were also bred to generate homozygous offspring. We first identified putative homozygotes by quantifying PCR amplicons that spanned the hTTR-tdTomato BAC. Specifically, we performed multiplex PCR reactions (two primer pairs per reaction) for JAX control mouse DNA and for either tdTomato coding sequence or flanking human TTR elements (Table 1). Three or four multiplex reactions were performed per mouse, subjected to gel electrophoresis (Fig. 3a), and analyzed by densitometry using ImageJ (Fig. 3b). The relative areas of the peaks corresponding to the BAC and control bands were calculated for each lane, and the ratios were plotted against those obtained for other sets of primer pairs (Fig. 3c). In these plots, individual mice typically clustered based on amplicon abundance (e.g. see mice 891 and 892 in red in Fig. 3). Mice with the most abundant BAC amplicons (i.e. putative homozygotes) were then bred with wild-type mice to test for homozygosity. Of the ten mice successfully bred, nine produced solely transgenic offspring (17–36 pups genotyped) and were deemed homozygous.

For founder line 3147, four homozygous males and two homozygous females were identified, and for founder line 3154, two homozygous females and one homozygous male were identified. All homozygous mice appeared normal, grew at indistinguishable rates from wild-type siblings, and successfully interbred to generate homozygous 3147 and 3154 lines, all offspring of which appear normal. For founder line 3161, 20 pups (three litters) from two hemizygous pairs were screened, but no putative homozygotes were identified; thus, no further attempts were made at achieving homozygosis of this line.

### Embryonic onset of tdTomato fluorescence in choroid plexus

**Fig. 4:**
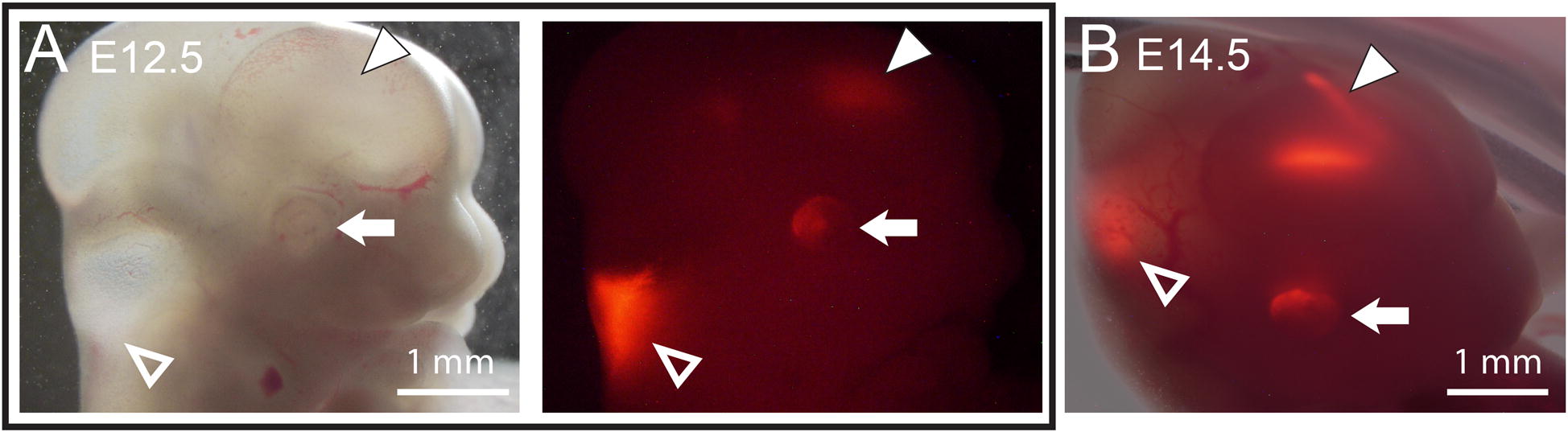
Expression of tdTomato in embryonic ChP is evident in images of fluorescence obtained through the intact head of embryos at E12.5 (**A**, right) and E14.5 (**B**). Note fluorescence in the hindbrain (open arrowheads), which is predominant at E12.5, as well as in the forebrain (solid arrowheads), which becomes more pronounced with the later development of the lateral ventricle ChP. Fluorescence is also evident in embryonic eyes (arrows)

Red fluorescence could be detected in heads of whole, unfixed embryos as early as E11.5. At E11.5 and E12.5, the greatest signal was detected in the hindbrain region (Fig. 4A, open arrowhead), which is consistent with the first appearance of the ChP [29, 30]. By E14.5, fluorescence in the forebrain had clearly taken on the bicornuate form of the two lateral ventricles (Fig. 4B, solid arrowhead), reflecting the development of the telencephalic choroid plexus.

We also detected tdTomato fluorescence in the eyes of whole, unfixed embryos as early as E12.5 (Fig. 4A, B, arrows). This fluorescence reflects transthyretin expression in retinal pigment epithelium, which will be discussed in more detail below.

### tdTomato is strongly expressed in choroid plexus throughout the postnatal period

**Fig. 5:**
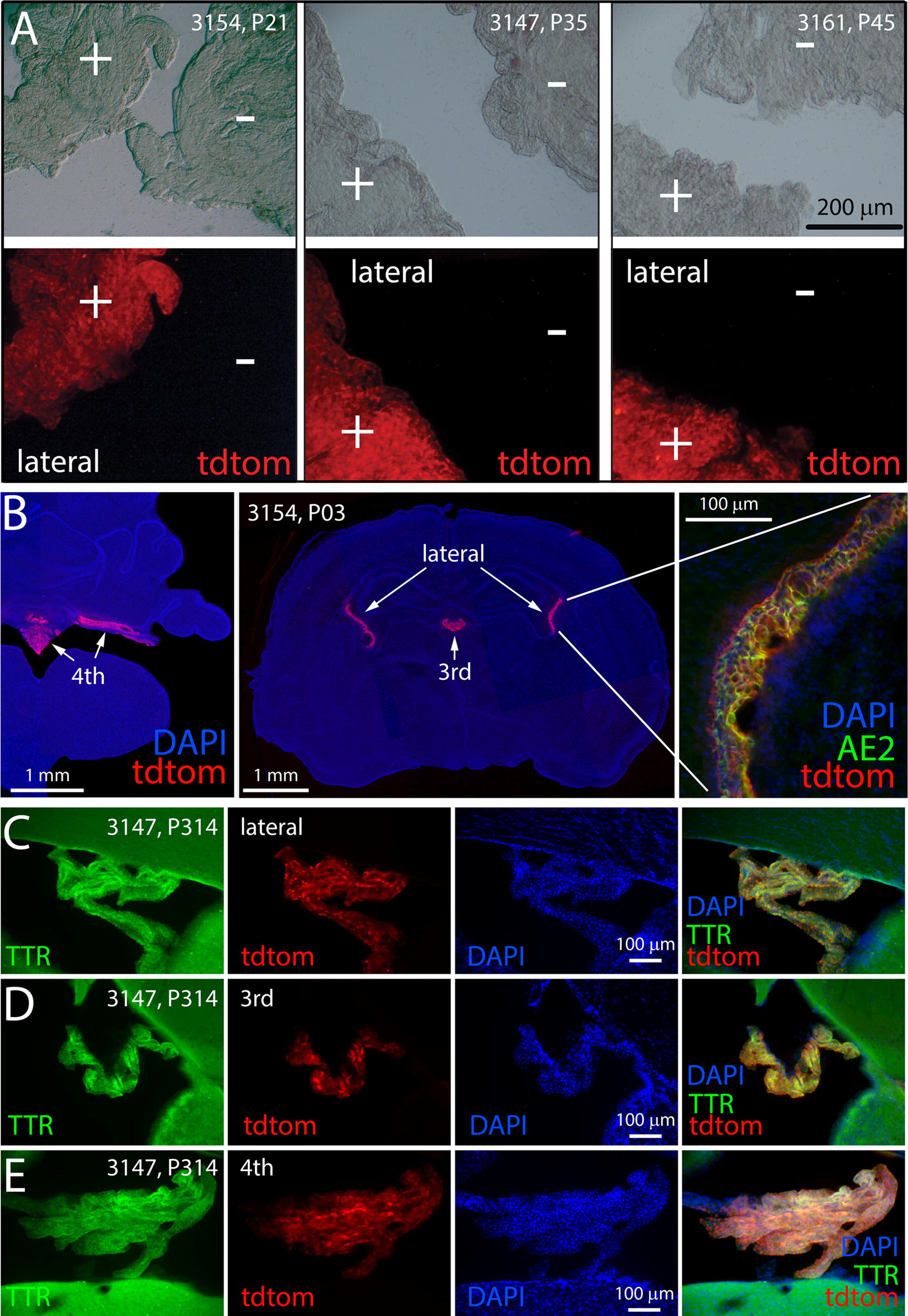
TdTomato continues to be expressed in postnatal choroid plexus of BAC transgenic mice. **A** Show paired images of transmitted light and tdTomato fluorescence in wet mounts of dissected lateral ventricle ChP from post-weanling mice from three founder lines. Whereas transgenic ChP (+) fluoresce brightly, ChP from wild-type siblings (−) do not. **B** Show immunofluorescence in cryostat sections from a P3 brain. TdTomato was detected in all four ventricles and was co-localized with anion exchanger 2 (AE2, right). **C**–**E** Shows immunofluorescence in microtome sections of brains from 314-day-old mice, verifying the long-term expression of the reporter in CPECs of the lateral (**C**), third (**D**), and fourth (**E**) ventricle. The selective localization of tdTomato in CPECs is confirmed by associated staining with antibodies to TTR. The location of nuclei is indicated by DAPI staining

We successfully imaged tdTomato in both unfixed and fixed ChP, with or without immunostaining, across a wide range of ages. Figure 5A shows images taken through a dissecting scope of whole mounts of unfixed lateral ventricle ChP dissected from weanling mice of three founder lines. The ChP of hemizygous mice (+) were bright from native tdTomato fluorescence, whereas the ChP from wild-type siblings that were imaged alongside (−) showed no fluorescence. All regions of the lateral and fourth ventricle ChP appeared to fluoresce, a strong contrast to the results with the previous mRFP1 reporter (Fig. 1).

We found that native tdTomato fluorescence photobleached rapidly, so we frequently chose to perform indirect immunofluorescence staining using an anti-RFP antibody (Rockland, 600-401-379) that reacts with tdTomato. TdTomato immunofluorescence was detected in all four ventricles of a P3 mouse (Fig. 5B) and was clearly present in the ChP and CPECs based on double-labeling with the CPEC marker AE2 (Fig. 5B, right panel). TdTomato expression remained robust in ChP from all ventricles at P314 (10.5 months of age; Fig. 5C–E), attesting to the longevity of tdTomato expression. Moreover, essentially every CPEC bearing TTR (green fluorescence) appeared also to express tdTomato (red fluorescence) in these images.

### TdTomato is expressed in a subset of cells in pancreatic islets

**Fig. 6:**
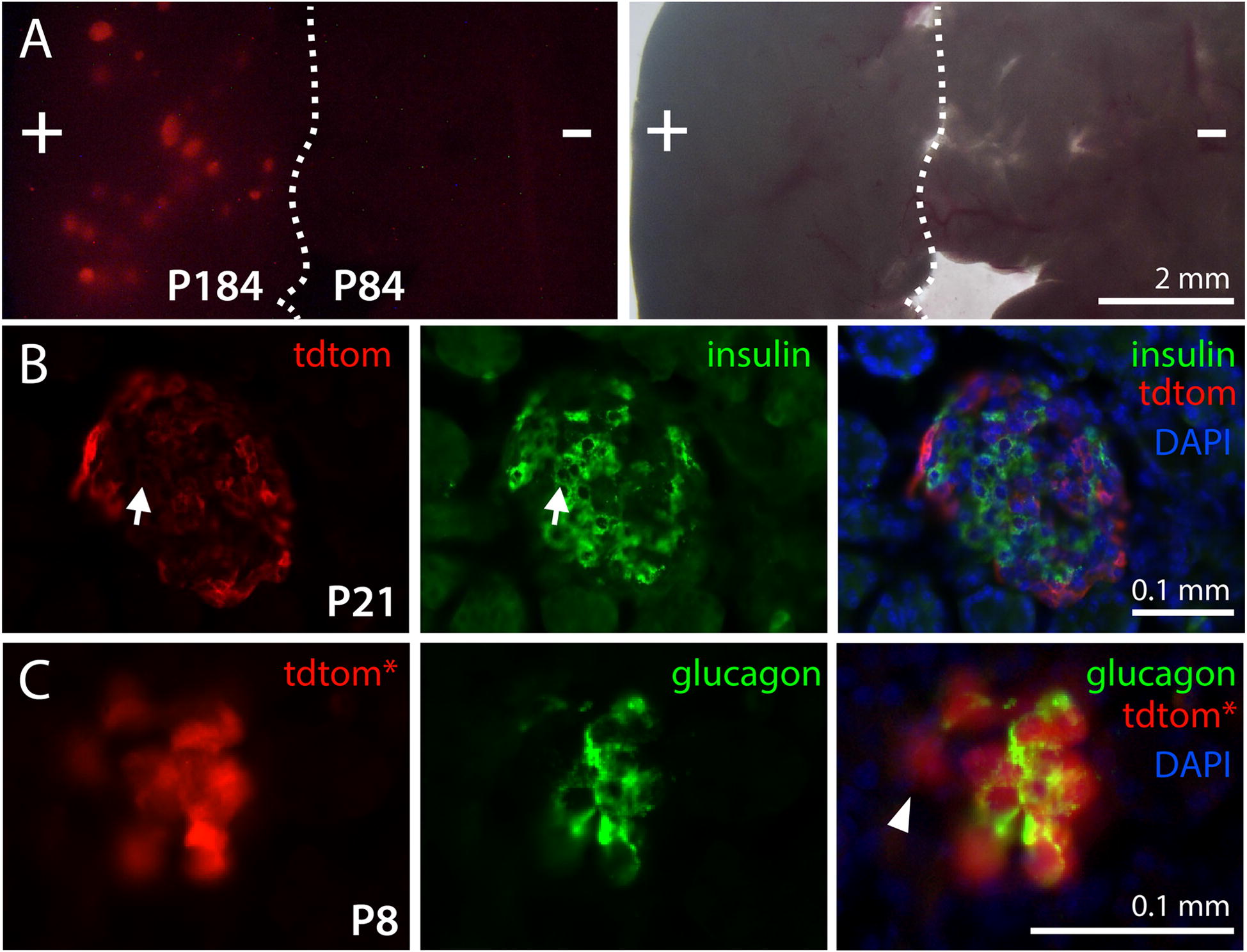
TdTomato is expressed in pancreatic islets. **A** Native tdTomato fluorescence in wet mounts of pancreas from a 6-month old transgenic mouse (+) compared to a wild-type mouse (−), with a corresponding transmitted light image at right. Fluorescence was observed in round or ellipsoid patches, consistent with expression in pancreatic islets. **B** Immunofluorescence in an islet from a 3-week old transgenic mouse. Cells expressing tdTomato immunoreactivity (tdtom) are largely distinct from beta cells expressing insulin, although some insulin-positive cells appear to be weakly stained for tdTomato (arrows). **C** Native tdTomato fluorescence (tdtom*) occurs in the same regions exhibiting immunostaining for glucagon in an islet from an 8-day old mouse, suggesting an association with alpha cells, although certain tdTomato-expressing cells may lack anti-glucagon staining (arrowhead)

TdTomato fluorescence was detected in pancreatic tissue from every transgenic mouse investigated, which included animals from P3 to 10.5 months of age. Figure 6A shows fluorescence in a wet mount of pancreas from a 6-month old transgenic mouse (+) alongside pancreas from a wild-type mouse (−). The observed patchy distribution of red fluorescence was consistent with the known expression of transthyretin in pancreatic islets [31]. In cryosections, some weak immunostaining for tdTomato more centrally within islets co-localized with insulin immunostains (arrows in Fig. 6B), suggesting low-level tdTomato expression in β-cells [31, 32], but more intense tdTomato staining occurred in the largely insulin-negative periphery of islets (Fig. 6B). At the periphery, bright native tdTomato fluorescence partially co-localized with immunostains for glucagon (Fig. 6C), consistent with TTR expression in α-cells [31–34]. However, peripheral tdTomato native fluorescence was also seen in glucagon-negative cells, suggesting the possibility of reporter expression in other non-α or -β islet cell types.

### TdTomato is highly expressed in retinal pigment epithelium (RPE)

**Fig. 7:**
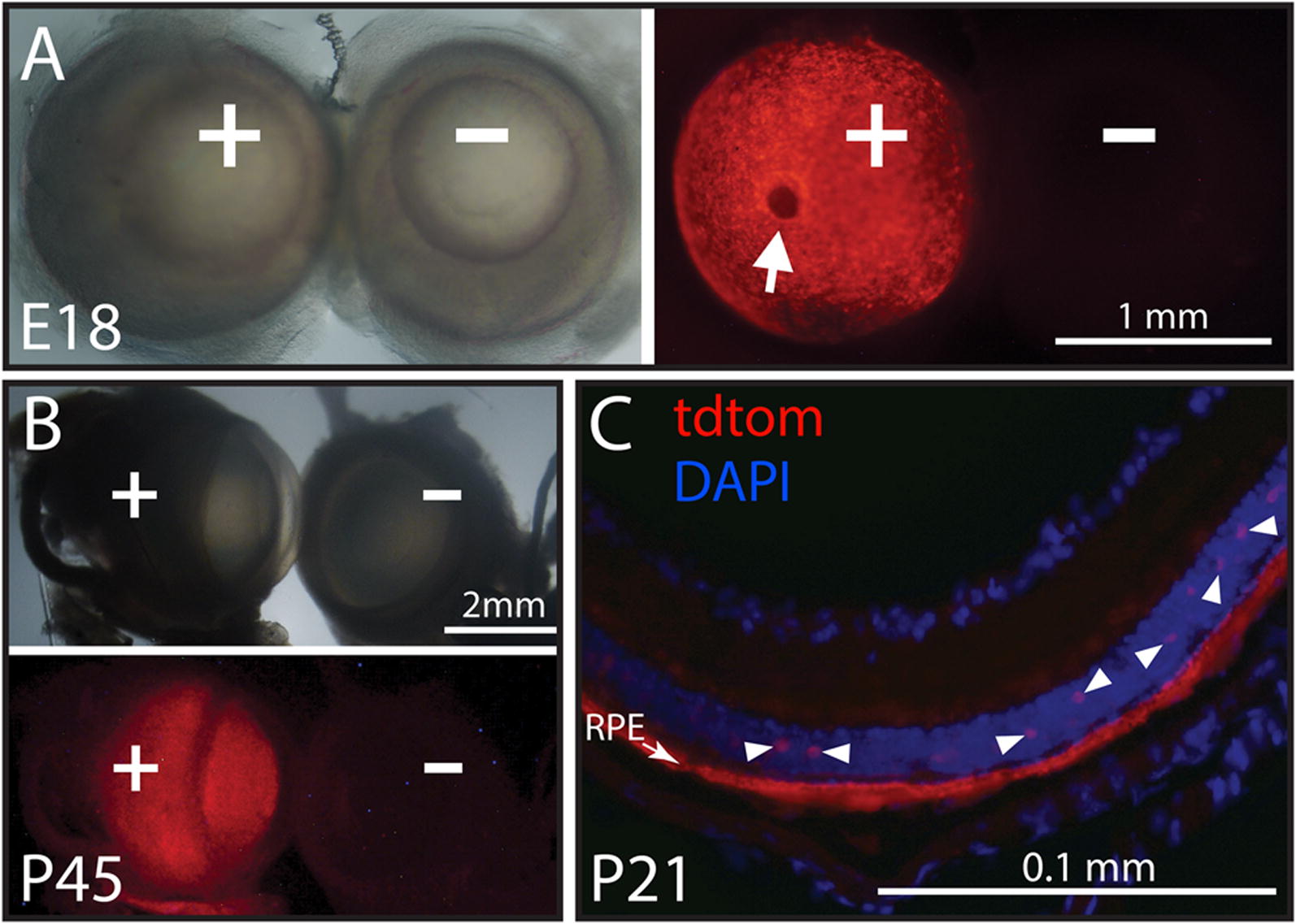
TdTomato is expressed in retinal pigment epithelium. **A** Shows corresponding brightfield (left) and red fluorescence (right) images of intact eyes dissected from a transgenic tdTomato E18 embryo (+) and a wild-type sibling (−). The back of the transgenic eye is fluorescent, except for the circular optic disk (arrow), where the retina is interrupted by outgoing axons of the optic nerve. **B** Shows similar images of eyes from a 1.5-month old pair of transgenic (+) and wild-type (−) siblings. **C** Shows immunofluorescence for tdTomato in a cryostat section taken transverse to the plane of the retina from a 3-week old transgenic mouse. The immunoreactive arc corresponds in location to the retinal pigment epithelium (RPE), known to express TTR. Intermittent immunostained large cells in the retinal layer were also observed (arrowheads)

**Fig. 8:**
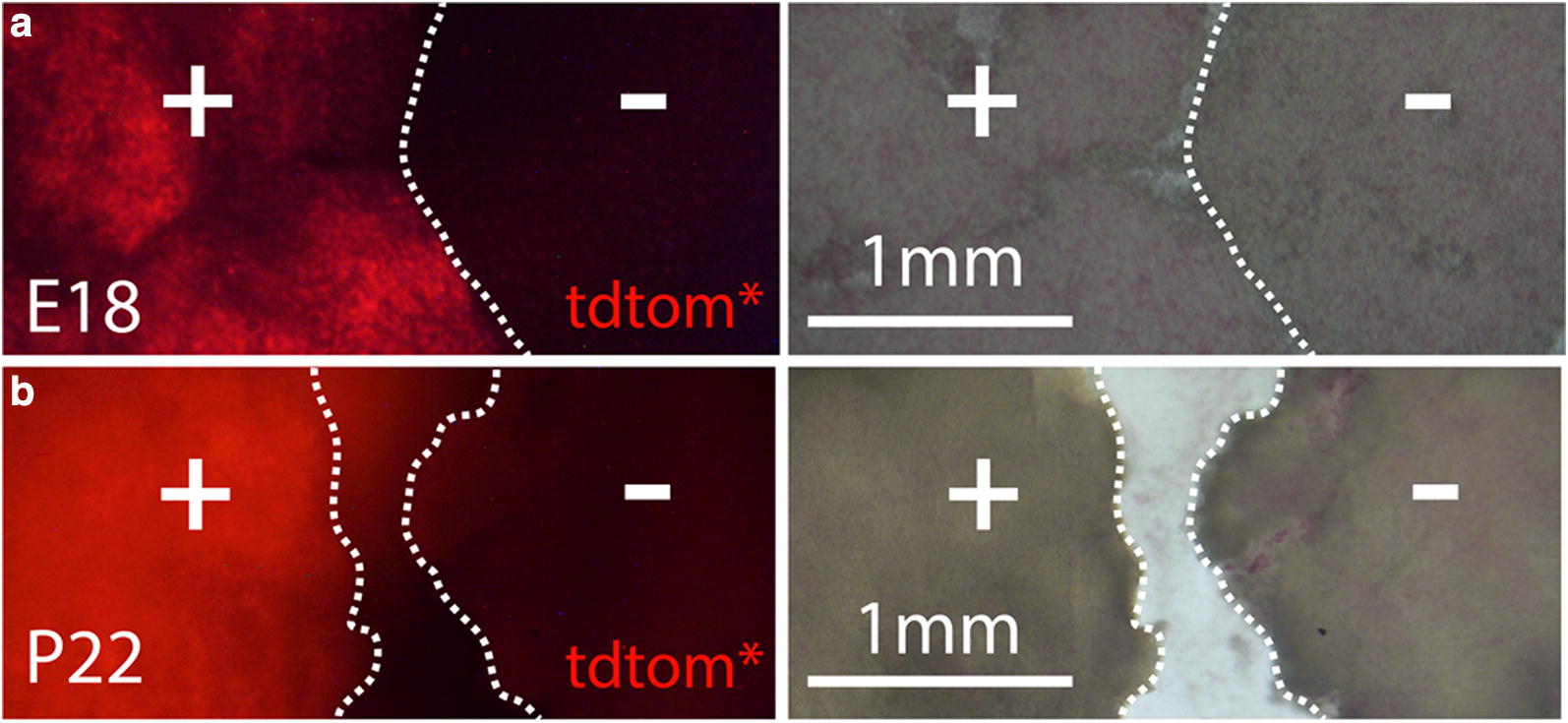
TdTomato is expressed in liver. Long photographic exposures of wet-mounted liver slices consistently revealed brighter native tdTomato fluorescence (tdtom*) in transgenic tissue (+) than in wild-type tissue (−). Shown are examples from an embryonic (**a**) and a weanling (**b**) mouse. Corresponding images of transmitted light are shown at right

**Fig. 9:**
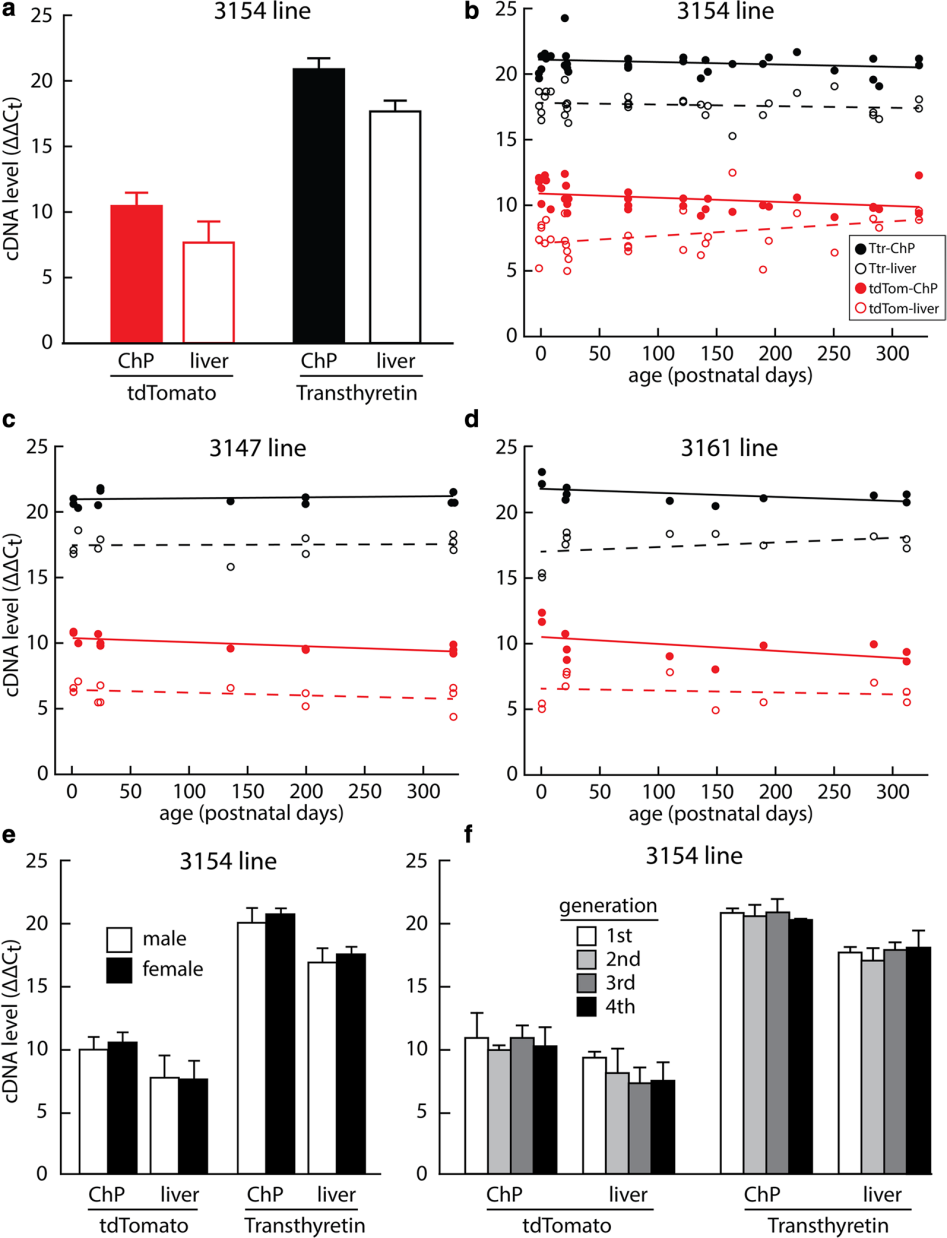
Real-time rt-qPCR revealed long-term expression of TTR and tdTomato mRNA. **a** Values across all animals from the 3154 founder line indicated a greater abundance of tdTomato transcript in the ChP than in the liver, and the difference was similar to that detected for transthyretin. **b** When expressed as a function of age, tdTomato transcripts in the ChP declined slightly but significantly in the 3154 founder line, whereas tdTomato transcripts in the liver increased significantly. In contrast, transthyretin transcripts remained steady with age in both ChP and liver. The small decline in tdTomato expression across age in the 3154 line was recapitulated in both the 3147 (**c**) and 3161 (**d**) founder lines, but the increase in tdTomato transcript in the liver was not observed for the 3147 and 3161 lines. Levels of transthyretin transcripts did not change significantly with age in either the ChP or liver in either the 3147 or 3161 founder line. TdTomato and transthyretin in the 3154 founder line did not differ between the sexes (**e**) or across generations (**f**). Heights of bars indicate mean values, error bars denote standard deviations, and trendlines are the results of linear regression

**Fig. 10:**
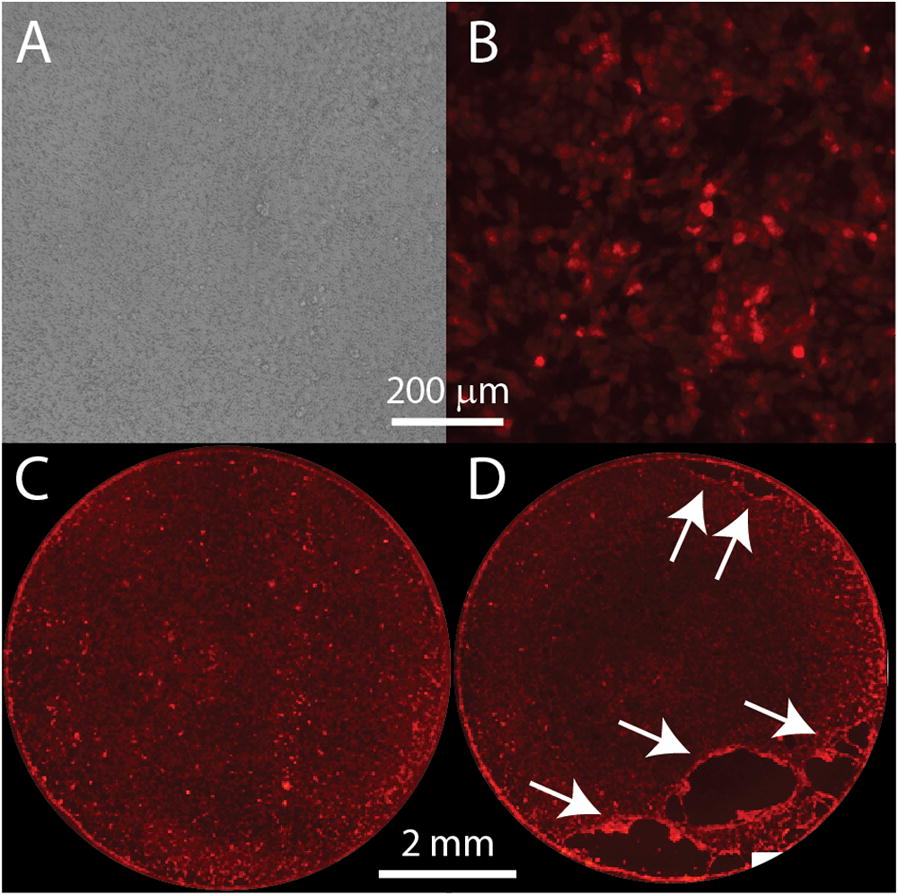
The tdTomato reporter facilitates visualization of cultured CPECs on Transwell membranes. **A** CPECs growing on Transwell membranes are not easily visible using phase contrast microscopy. **B** When visualized using red epifluorescence, the same field as in **A** could be seen to possess a confluent monolayer of primary CPECs originally dissociated from the ChP of homozygous TTR BAC-tdTomato transgenic mice (3147 founder line). **C** Stitched images of tdTomato fluorescence revealed nearly 100% coverage of the Transwell membrane after 4 days of undisturbed culture. The monolayer displayed a respectable trans-epithelial electrical resistance of 110 Ω cm^2^. **D** The same membrane as in **C** was imaged after 3 days of experimental manipulation. Holes in the coverage were clearly evident (arrows), explaining a measure decrease of resistance to 44 Ω cm^2^

TdTomato fluorescence was also detected in eyes from every transgenic mouse investigated from E12.5 (Fig. 4) to 10 months of age and from all three transgenic lines. Figure 7A shows an image looking down through the lens of an intact, unfixed E18.5 transgenic eye (+) alongside an eye from a wild-type sibling (−). Most of the transgenic retina fluoresced, with the exception of the optic disk where retina is normally absent (arrow). Figure 7B shows a side view of eyes from P45 transgenic (+) and wild-type siblings (−), illustrating the persistence of fluorescence into the second postnatal month. In cryosections, immunostaining for tdTomato was most intense in the RPE (Fig. 7C), consistent with the known TTR expression in that layer [35, 36]. Apparent staining of sparsely distributed cells in the retina was also observed (arrowheads), which may represent horizontal or amacrine cells, although TTR mRNA detected by in situ hybridization has been reported to be absent outside of the RPE in the rat eye [34].

### TdTomato fluorescence is detectable in the liver

Transthyretin is well known to be synthesized by hepatocytes, the main source of TTR in plasma [37]. Accordingly, tdTomato fluorescence was detected in wet mounts of thin liver slices taken from transgenic mice ranging in age from E18 through P22 (Fig. 8). However, longer exposures were invariably required to detect fluorescence compared to the previously described tissues, and comparisons to wild-type liver slices were essential to distinguish signal definitively from autofluorescence.

### Real time RT-qPCR confirms long-term expression of tdTomato

To evaluate whether tdTomato expression accurately reports TTR expression, we used real-time RT-qPCR to quantify relative levels of tdTomato and TTR mRNA in ChP and liver in hemizygous mice. Average Ct values for 18S rRNA and cyclophilin mRNA together were used as reference standards to calculate ΔCt values. The RT-qPCR assay clearly detected tdTomato mRNA, as ΔCt values for hemizygous mice (n = 56) differed significantly from values for wild-type mice (n = 24) in both tissues (ChP: hemizygous ΔCt = − 11.1 ± 1.5 (s.d.), wild type ΔCt = − 20.5 ± 3.8, two-tailed t = 15.7, p = 8.5 × 10^−26^; liver: hemizygous ΔCt = − 14.8 ± 1.7, wild type-21.9 ± 2.7, t = 14.2, p = 2.2 × 10^−23^). We also measured levels of TTR, which did not differ between hemizygous and wild-type mice (ChP: hemizygous ΔCt = − 0.2 ± 0.8, wild type ΔCt = − 0.2 ± 1.1, t = 0.05, p = 0.96; liver: hemizygous ΔCt = − 4.2 ± 0.9, wild type ΔCt = − 4.0 ± 1.0, t = − 0.99, p = 0.32). The fact that levels of TTR mRNA did not differ between transgenic and wild-type mice indicates that the reporter sequence did not interfere significantly with transcription of the endogenous TTR gene.

Figure 9a shows the ΔΔCt values obtained for ChP and liver from all mice we investigated from the 3154 founder line (n = 32 hemizygotes). Consistent with the brighter native tdTomato fluorescence of the ChP compared to the liver, RT-qPCR showed that ChP expressed greater levels of both tdTomato and TTR transcripts than did liver. The ChP-liver difference was also similar for the two transcripts [2.8 ± 1.9 (s.d.) Ct values for tdTomato and 3.2 ± 0.9 Ct values for TTR, paired *t* test: t = − 0.94, p = 0.35], corresponding to ~ 8-fold higher transcript levels in ChP than in liver. The fact that ChP and liver displayed similar differences in transcription of tdTomato and TTR implies that the human TTR regulatory sequences in the BAC are similarly interpreted by the mouse transcription factors that specify tissue dependence and levels.

Figure 9b shows the ΔΔCt values from the 3154 founder line as a function of mouse age. Whereas TTR displayed no significant differences with age in either the ChP or the liver, regression analysis indicated a small, but statistically significant, age-related decline in tdTomato transcripts in the ChP (− 0.0036 Ct value/day, r = 0.40, F = 5.78, p = 0.022) and a significant but small age-related increase in the liver (0.0055 Ct value/day, r = 0.37, F = 4.66, p = 0.039).

There were no significant differences in ChP tdTomato ΔΔCt values across the three founder lines that were chosen for detailed analysis (single-factor analysis of variance (ANOVA) F_(2,53)_ = 0.34, p = 0.72); however, tdTomato ΔΔCt values in liver did show significant differences (one-way ANOVA F_(2,53)_ = 4.59, p = 0.016). Post-hoc t-tests revealed a significant difference between the 3154 line and the 3147 line (t = 2.49, p = 0.016), while the difference between 3154 and 3161 lines did not reach the significance threshold (p = 0.059). The slight age-dependent decline in tdTomato ΔΔCt values in the ChP in the 3154 line (Fig. 9b) was also significant for the 3147 line, despite being assessed with fewer data points (− 0.0030 Ct value/day, r = 0.72, F = 11.0, p = 0.0077) (Fig. 9c). A similar trend towards age-related decline was apparent for the 3161 line (Fig. 9d), but was not found to be significant (r = 0.49, F = 2.80, p = 0.13). Unlike the 3154 line, however, the 3147 and 3161 founder lines did not show any age-dependent increase in liver tdTomato transcripts (Fig. 9c, d). Similar to the 3154 line, TTR transcripts did not change significantly with age in either 3147 or 3161 founder lines (Fig. 9c, d). The fact that all three founder lines showed a slight age-related tdTomato decline in the ChP, while TTR transcripts remained unchanged, suggests the possibility of a human-mouse difference in TTR regulation that is independent of integration site.

Real-time RT-qPCR further indicated that mRNA levels for tdTomato and TTR did not differ between sexes in either tissue of the 3154 founder line (Fig. 9e, n = 15 males and 9 females), consistent with prior experiments showing no sex difference in TTR transcripts in the mouse liver [38]. Figure 9f shows that there was no consistent decline in tdTomato ΔΔCt values over four successive generations in the 3154 founder line, suggesting stability of the BAC transgene and its expression in the ChP and liver. (The liver values were not statistically different: single-factor ANOVA F_(3,28)_ = 1.29, p = 0.30).

Thus, the real-time RT-qPCR data indicates that the human TTR BAC tdTomato reporter largely parallels the expression of the endogenous mouse TTR gene in terms of ChP and liver expression profiles in a fashion that is gender-independent, integration site-independent, and stable across generations, with consistently higher levels of TTR and tdTomato in ChP compared to liver. There were, however, small but significant age-related declines in ChP transcription of tdTomato across lines that were not observed for TTR.

### The TTR BAC-tdTomato reporter is useful in characterizing monolayer cultures of CPECs grown in transwell chambers

Primary monolayer cultures of CPECs in Transwell chambers are useful for studying the barrier and transport functions of the ChP [22, 39–41]. It would be beneficial to monitor the coverage of the Transwell membrane by these live cells over time and across different experimental conditions, but the cells can be difficult to visualize by phase contrast microscopy because of the strong pattern from the porous membrane upon which they are grown (Fig. 10A). Fortunately, primary Transwell cultures derived from homozygous TTR BAC-tdTomato mice could be easily visualized by way of their red fluorescence (Fig. 10B). Furthermore, the presence of fluorescence throughout the cytoplasm of these cells supports an evaluation of the confluence of cells when the entire membrane is imaged by way of stitching together spatially-ordered snapshots of a culture (Fig. 10C). The ability to evaluate confluence non-invasively has aided in the interpretation of the loss of barrier function sometimes observed after having performed multiple experiments on the same culture over time. For example, the culture shown in Fig. 10C displayed a healthy trans-epithelial electrical resistance of 110 Ω cm^2^, whereas a drop in the value to 44 Ω cm^2^ observed after 2 days of experiments was clearly associated with a detached monolayer when tdTomato fluorescence was evaluated (Fig. 10D).

### Generation of human TTR BAC-luc2 reporter mice

A total of 11 mice were born from eggs injected with the luc2 reporter BAC. (See “Methods” for details.) PCR showed that five of these mice possessed both internal sequences of luc2 cDNA and human BAC sequences flanking the reporter (Fig. 2). Two of the positive mice were bred to test for expression of luciferase activity in various tissues of their progeny. Both of these strains have been archived by sperm cryopreservation, and the sperm has been verified for fertility after thawing.

### Long-term persistence of luciferase activity in TTR-expressing tissues of transgenic mice

**Fig. 11:**
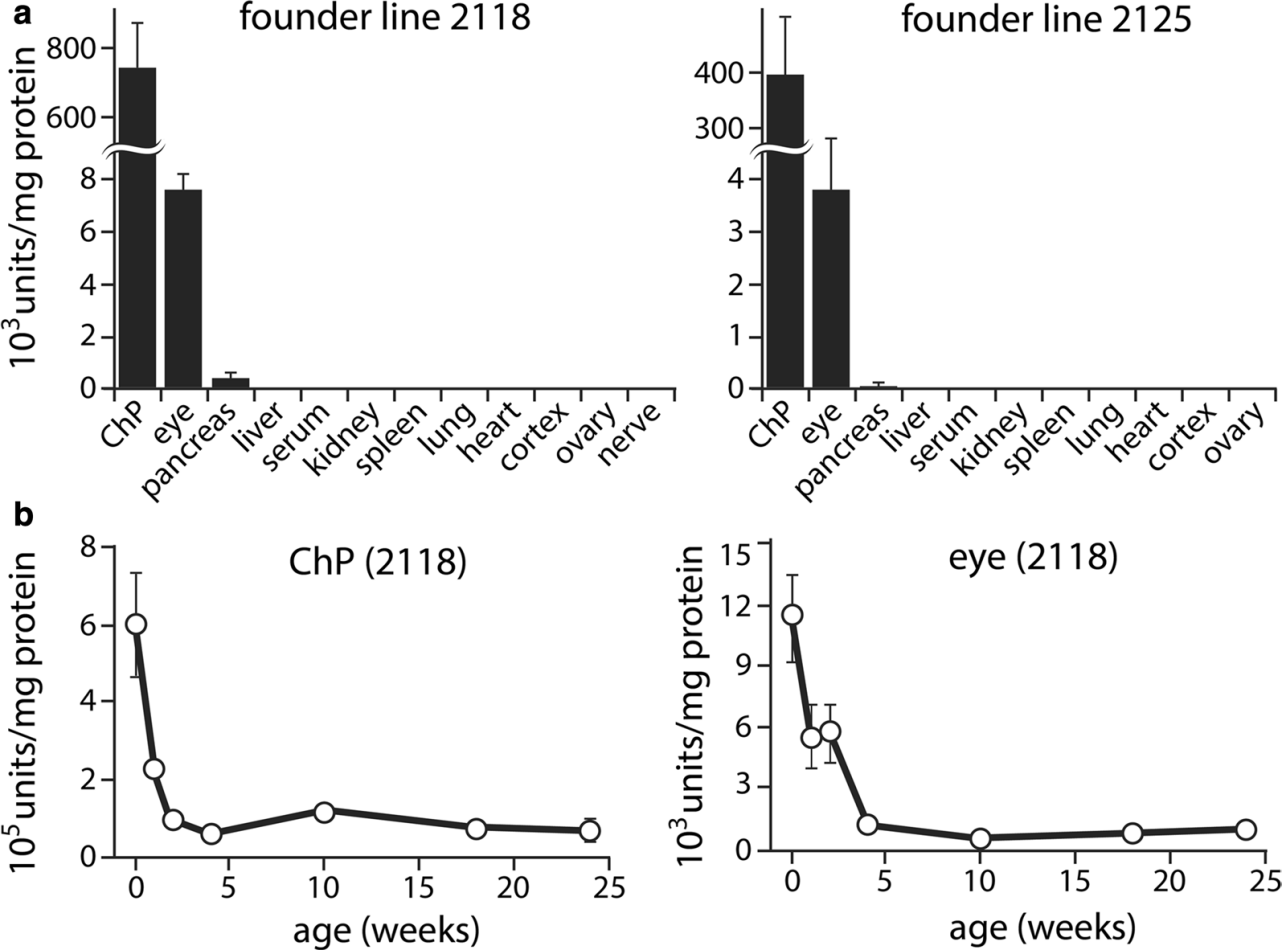
Luciferase activity is high in the ChP of transgenic mice. Luciferase activity measured in lysates of various tissues in two different founder lines showed a rank order largely consistent with the intensity of tdTomato native fluorescence, with no detection of luciferase in tissues that do not express TTR (**a**). The specific activity of luciferase in both the ChP and the eye declined rapidly from high perinatal values to steadily detectable levels that persisted well into adulthood (**b**). Error bars denote standard deviations

The tissue distribution of specific luciferase activity was very similar between the two founder lines, although line 2118 had twice the activity of line 2125 (Fig. 11a). Of the tissues examined, ChP had by far the greatest specific activity, 100 times that of the eye, the second most active tissue. Activity was low, but detectable in the pancreas, but was not detected in the liver. The presence of luc2 activity in ChP, eye, and pancreas is consistent with the tdTomato reporter and the known expression of TTR, while the absence of activity in liver was surprising given the tdTomato reporter data (Figs. 8 and 9), but was consistent for the two independent luc2 founder lines.

When luciferase specific activity in the ChP and eye were followed as a function of age in tissue from founder line 2118, very high perinatal activity in both tissues was found to decline rapidly to lower, but still easily detectable, levels that then remained stable from 2 weeks to 6 months of age (Fig. 11b). Thus, the sustained expression of the luciferase reporter and the rank order of tissue expression were similar to those of the tdTomato reporter, and the consistency across the two luc2 lines recalls the similarity in expression across the three tdTomato founder lines that we studied.

## Discussion

We have demonstrated accurate reporting of transthyretin expression in transgenic mice expressing tdTomato fluorescent protein by way of randomly integrated human TTR BACs. The major tissues in which TTR is expressed postnatally and constitutively (choroid plexus, liver, retinal pigment epithelium, and pancreatic islets) all displayed endogenous red fluorescence when compared to wild type controls, and no ectopic expression was detected. Quantitative evaluation of transcription by rt-qPCR showed that relative expression of tdTomato in the liver and ChP paralleled relative expression of TTR in those two tissues. No sex differences were detected, and expression was maintained over successive generations. Appropriate cellular specificity was documented by immunostaining using antibodies that recognize the tdTomato protein. Expression of tdTomato in the embryonic choroid plexus coincided with the known onset of TTR expression [29, 30, 42], and ChP tdTomato expression persisted far into adulthood. Similarity across three independent founder lines indicated that the characteristics of expression were independent of integration site, a typical advantage of BAC transgenes [18]. Activity of luc2 was also similar in two independent reporter lines generated from the human TTR BACs.

A prior TTR::RFP reporter transgenic mouse [17] was based on a TTR minigene involving 3kbp of DNA upstream of the mouse TTR coding sequence, a region that had been shown in prior studies to contain the positive elements required for ChP expression [43, 44]. Despite expression of this minigene in the ChP, ectopic expression in brain regions other than the ChP was also observed [17, 43], and embryonic expression of RFP and Cre were detected in regions of the limb that have not previously been associated with TTR expression [17]. As previously mentioned, expression of the RFP reporter was mosaic in the ChP and disappeared from the lateral and third ventricle in the first few postnatal weeks. These anomalies could reflect the absence of important regulatory elements from the 3-kbp portion of the mouse gene, or they could reflect positional effects on expression caused by the site of integration in the mouse chromosome [18].

Positive elements in the human TTR gene that are required for expression of a mutant TTR in transgenic mouse ChP were found to be within 6 kbp upstream of the TTR coding sequence [45]. The presence of these regulatory sequences in the human TTR BAC, as well as the very large expanse of DNA from the BAC that would tend to obviate positional effects, may explain the more faithful expression of tdTomato in the human TTR BAC transgenic mice.

In the 6 kbp mutant human TTR transgenic mouse, it was noted that levels of liver transcripts for the mutant TTR increased with age following birth, while postnatal levels of the native mouse TTR transcript remained constant following an embryonic increase, and it was suggested that the postnatal increase in human TTR may reflect a difference in the regulation of the human TTR gene in the mouse liver [45]. In one of our founder lines (3154), we also observed a postnatal increase in transcription of tdTomato in the liver; however, this increase was not observed in the other two founder lines. On the other hand, all three founder lines we investigated displayed a small postnatal decrease in transcription of the tdTomato reporter in the ChP over the course of the first postnatal year, while levels of the native mouse TTR transcript in ChP remained constant, which may be evidence of a difference in regulation of the human and mouse genes in the ChP of transgenic mice. Although we found no changes in the level of the native mouse TTR transcript over the course of the first postnatal year, there has been one report of a decrease in CSF TTR protein levels in 18-month-old mice [46]. We cannot rule out the possibility that we would have detected declines in ChP TTR transcripts had we investigated such older mice.

The TTR reporters developed here should facilitate future studies of CPEC biology. In addition to the enhanced ability to visualize and analyze CPECs in Transwell cultures that we have demonstrated in the current paper, the reporters should provide tools to track CPECs following transplantation, to purify CPECs for detailed study and manipulation, and to report on CPEC derivation from stem cells. TTR reporters may be similarly useful in studying retinal pigment epithelial cells, pancreatic islet cells, and hepatocytes. In addition to these important uses, the myriad functions being revealed for TTR [9, 47] suggest that these reporters may enjoy a broader set of applications. Originally known as prealbumin, transthyretin was renamed for its ability to transport thyroxine and retinol binding protein; now, TTR is known to bind to numerous other proteins and to function as a protease, cleaving such important substrates as beta-amyloid peptide [48], apolipoprotein AI [49], and neuropeptide Y [50]. The ability of TTR to fragment the Alzheimer’s disease-related beta-amyloid peptide recently has been reported to be central to its ability to decrease the toxicity of this peptide [51], and TTR also participates in the transport of beta-amyloid out of the brain to the liver [52]. TTR is expressed in neurons during heat shock and during the progression of mouse models of familial Alzheimer’s disease [16, 24]. TTR also binds to cell surface receptors directly and independently of binding thyroxine or retinol binding protein to promote nerve regeneration [53] and to affect synthesis of metabolic enzymes in astrocytes [54]. Aggregates of mutated and wild type TTR are responsible for various TTR amyloidoses, and treatment options currently being tested include genetic strategies to knock down synthesis of the protein [25]. Transgenic mice bearing reporters of TTR synthesis should be particularly useful for studying responses of the cell types involved in these myriad functions as well as to screen drugs to enhance or interfere with synthesis of TTR. The presence of human regulatory sequences in the BAC transgenes should increase the biomedical relevance of such studies.

The consistency of reporter expression across multiple founder lines suggests that TTR BACs may prove to be a reliable means to overexpress, knock down (via regulatory RNA expression), or knockout (via Cre recombinase expression and matings with floxed null mice) expression of other proteins in CPECs and other TTR-expressing cells.

## Conclusions

Transgenic mice bearing fluorescent and luminescent reporters of transthyretin have been developed and characterized. We have found that reporter expression profiles match those expected for transthyretin, and they remain expressed into adulthood. These reporter mice should prove useful for tracking transplanted choroid plexus epithelial cells, for purifying the cells for further study, and for reporting their derivation from stem cells. They also should prove useful for studying transthyretin synthesis by other cell types, as transthyretin has been implicated in many functions and conditions, including clearance of β-amyloid peptides associated with Alzheimer’s disease, heat shock in neurons, processing of neuropeptides, nerve regeneration, astrocyte metabolism, and transthyretin amyloidosis.

## Abbreviations

AE2: anion-exchange protein 2
ANOVA: analysis of variance
BAC: bacterial artificial chromosome
ChP: choroid plexus
CPEC: choroid plexus epithelial cell
CSF: cerebrospinal fluid
PBS: phosphate-buffered saline
RFP: red fluorescent protein
RPE: retinal pigment epithelium
RT-qPCR: reverse transcription quantitative polymerase chain reaction
TTR: transthyretin

## References

[1] Lehtinen MK, Bjornsson CS, Dymecki SM, Gilbertson RJ, Holtzman DM, Monuki ES (2013). The choroid plexus and cerebrospinal fluid: emerging roles in development, disease, and therapy. J Neurosci.

[2] Spector R, Keep RF, Robert Snodgrass S, Smith QR, Johanson CE (2015). A balanced view of choroid plexus structure and function: focus on adult humans. Exp Neurol.

[3] Wright BL, Lai JT, Sinclair AJ (2012). Cerebrospinal fluid and lumbar puncture: a practical review. J Neurol.

[4] Angelow S, Galla HJ, Zheng W, Chodobski (2005). Junctional proteins of the blood-CSF barrier. The blood-cerebrospinal fluid barrier.

[5] Brown PD, Speake T, Davies SL, Millar ID, Zheng W, Chodobski (2005). Ion transporters and channels involved in CSF formation. The blood-cerebrospinal fluid barrier.

[6] Miller DS, Lowes S, Pritchard JB, Zheng W, Chodobski (2005). The molecular basis of xenobiotic transport and metabolism. The blood-cerebrospinal fluid barrier.

[7] Serot JM, Béné MC, Faure GC (2003). Choroid plexus, aging of the brain, and Alzheimer’s disease. Front Biosci.

[8] Emerich DF, Skinner SJ, Borlongan CV, Vasconcellos AV, Thanos CG (2005). The choroid plexus in the rise, fall and repair of the brain. BioEssays.

[9] Fleming CE, Nunes AF, Sousa MM (2009). Transthyretin: more than meets the eye. Prog Neurobiol.

[10] Reboldi A, Coisne C, Baumjohann D, Benvenuto F, Bottinelli D, Lira S, Uccelli A, Lanzavecchia A, Engelhardt B, Sallusto F (2009). C-C chemokine receptor 6-regulated entry of TH-17 cells into the CNS through the choroid plexus is required for the initiation of EAE. Nat Immunol.

[11] Zhang X, Wu C, Song J, Götte M, Sorokin L (2013). Syndecan-1, a cell surface proteoglycan, negatively regulates initial leukocyte recruitment to the brain across the choroid plexus in murine experimental autoimmune encephalomyelitis. J Immunol.

[12] Watanabe M, Kang YJ, Davies LM, Meghpara S, Lau K, Chung CY, Kathiriya J, Hadjantonakis AK, Monuki ES (2012). BMP4 sufficiency to induce choroid plexus epithelial fate from embryonic stem cell-derived neuroepithelial progenitors. J Neurosci.

[13] Marques F, Sousa JC, Coppola G, Gao F, Puga R, Brentani H, Geschwind DH, Sousa N, Correia-Neves M, Palha JA (2011). Transcriptome signature of the adult mouse choroid plexus. Fluids Barriers CNS.

[14] Lun MP, Johnson MB, Broadbelt KG, Watanabe M, Kang YJ, Chau KF, Springel MW, Malesz A, Sousa AM, Pletikos M, Adelita T, Calicchio ML, Zhang Y, Holtzman MJ, Lidov HG, Sestan N, Steen H, Monuki ES, Lehtinen MK (2015). Spatially heterogeneous choroid plexus transcriptomes encode positional identity and contribute to regional CSF production. J Neurosci.

[15] Richardson SJ (2007). Cell and molecular biology of transthyretin and thyroid hormones. Int Rev Cytol.

[16] Wang X, Cattaneo F, Ryno L, Hulleman J, Reixach N, Buxbaum JN (2014). The systemic amyloid precursor transthyretin (TTR) behaves as a neuronal stress protein regulated by HSF1 in SH-SY5Y human neuroblastoma cells and APP23 Alzheimer’s disease model mice. J Neurosci.

[17] Kwon GS, Hadjantonakis AK (2009). Transthyretin mouse transgenes direct RFP expression or Cre-mediated recombination throughout the visceral endoderm. Genesis.

[18] Yang XW, Gong S (2005). An overview on the generation of BAC transgenic mice for neuroscience research. Curr Protoc Neurosci.

[19] Chan W, Costantino N, Li R, Lee SC, Su Q, Melvin D, Court DL, Liu P (2007). A recombineering based approach for high-throughput conditional knockout targeting vector construction. Nucleic Acids Res.

[20] Wang S, Zhao Y, Leiby M, Zhu J (2009). A new positive/negative selection scheme for precise BAC recombineering. Mol Biotechnol.

[21] Vandesompele J, De Preter K, Pattyn F, Poppe B, Van Roy N, De Paepe A, Speleman F (2002). Accurate normalization of real-time quantitative RT-PCR data by geometric averaging of multiple internal control genes. Genome Biol..

[22] Monnot AD, Zheng W (2013). Culture of choroid plexus epithelial cells and in vitro model of blood-CSF barrier. Methods Mol Biol.

[23] Costa R, Gonçalves A, Saraiva MJ, Cardoso I (2008). Transthyretin binding to A-Beta peptide–impact on A-Beta fibrillogenesis and toxicity. FEBS Lett.

[24] Li X, Buxbaum JN (2011). Transthyretin and the brain re-visited: is neuronal synthesis of transthyretin protective in Alzheimer’s disease?. Mol Neurodegener.

[25] Sekijima Y (2015). Transthyretin (ATTR) amyloidosis: clinical spectrum, molecular pathogenesis and disease-modifying treatments. J Neurol Neurosurg Psychiatry.

[26] Alemi M, Silva SC, Santana I, Cardoso I (2017). Transthyretin stability is critical in assisting beta amyloid clearance—relevance of transthyretin stabilization in Alzheimer’s disease. CNS Neurosci Ther.

[27] Shaner NC, Campbell RE, Steinbach PA, Giepmans BN, Palmer AE, Tsien RY (2004). Improved monomeric red, orange and yellow fluorescent proteins derived from *Discosoma* sp. red fluorescent protein. Nat Biotechnol.

[28] Paguio A, Almond B, Fan F, Stecha P, Garvin D, Wood M, Wood K (2005). pGL4 vectors: a new generation of luciferase reporter vectors. Promega Notes..

[29] Dziegielewska KM, Ek J, Habgood MD, Saunders NR (2001). Development of the choroid plexus. Microsc Res Tech.

[30] Currle DS, Cheng X, Hsu CM, Monuki ES (2005). Direct and indirect roles of CNS dorsal midline cells in choroid plexus epithelia formation. Development.

[31] Jacobsson B, Collins VP, Grimelius L, Pettersson T, Sandstedt B, Carlström A (1989). Transthyretin immunoreactivity in human and porcine liver, choroid plexus, and pancreatic islets. J Histochem Cytochem.

[32] Dorrell C, Grompe MT, Pan FC, Zhong Y, Canaday PS, Shultz LD, Greiner DL, Wright CV, Streeter PR, Grompe M (2011). Isolation of mouse pancreatic alpha, beta, duct and acinar populations with cell surface markers. Mol Cell Endocrinol.

[33] Westermark GT, Westermark P (2008). Transthyretin and amyloid in the islets of Langerhans in type-2 diabetes. Exp Diabetes Res..

[34] Su Y, Jono H, Misumi Y, Senokuchi T, Guo J, Ueda M, Shinriki S, Tasaki M, Shono M, Obayashi K, Yamagata K, Araki E, Ando Y (2012). Novel function of transthyretin in pancreatic alpha cells. FEBS Lett.

[35] Cavallaro T, Martone RL, Dwork AJ, Schon EA, Herbert J (1990). The retinal pigment epithelium is the unique site of transthyretin synthesis in the rat eye. Invest Ophthalmol Vis Sci.

[36] Dwork AJ, Cavallaro T, Martone RL, Goodman DS, Schon EA, Herbert J (1990). Distribution of transthyretin in the rat eye. Invest Ophthalmol Vis Sci.

[37] Felding P, Fex G (1982). Cellular origin of prealbumin in the rat. Biochim Biophys Acta.

[38] Gonçalves I, Alves CH, Quintela T, Baltazar G, Socorro S, Saraiva MJ, Abreu R, Santos CR (2008). Transthyretin is up-regulated by sex hormones in mice liver. Mol Cell Biochem.

[39] Lazarevic I, Engelhardt B (2016). Modeling immune functions of the mouse blood-cerebrospinal fluid barrier in vitro: primary rather than immortalized mouse choroid plexus epithelial cells are suited to study immune cell migration across this brain barrier. Fluids Barriers CNS..

[40] Baehr C, Reichel V, Fricker G (2006). Choroid plexus epithelial monolayers—a cell culture model from porcine brain. Cerebrospinal Fluid Res..

[41] Hakvoort A, Haselbach M, Galla HJ (1998). Active transport properties of porcine choroid plexus cells in culture. Brain Res.

[42] Murakami T, Yasuda Y, Mita S, Maeda S, Shimada K, Fujimoto T, Araki S (1987). Prealbumin gene expression during mouse development studied by in situ hybridization. Cell Differ..

[43] Yan C, Costa RH, Darnell JE, Chen JD, Van Dyke TA (1990). Distinct positive and negative elements control the limited hepatocyte and choroid plexus expression of transthyretin in transgenic mice. EMBO J.

[44] Costa RH, Van Dyke TA, Yan C, Kuo F, Darnell JE (1990). Similarities in transthyretin gene expression and differences in transcription factors: liver and yolk sac compared to choroid plexus. Proc Natl Acad Sci USA.

[45] Nagata Y, Tashiro F, Yi S, Murakami T, Maeda S, Takahashi K, Shimada K, Okamura H, Yamamura K (1995). A 6-kb upstream region of the human transthyretin gene can direct developmental, tissue-specific, and quantitatively normal expression in transgenic mouse. J Biochem.

[46] Sousa JC, Marques F, Dias-Ferreira E, Cerqueira JJ, Sousa N, Palha JA (2007). Transthyretin influences spatial reference memory. Neurobiol Learn Mem.

[47] Buxbaum JN, Reixach N (2009). Transthyretin: the servant of many masters. Cell Mol Life Sci.

[48] Costa R, Ferreira-da-Silva F, Saraiva MJ, Cardoso I (2008). Transthyretin protects against A-beta peptide toxicity by proteolytic cleavage of the peptide: a mechanism sensitive to the Kunitz protease inhibitor. PLoS ONE.

[49] Liz MA, Faro CJ, Saraiva MJ, Sousa MM (2004). Transthyretin, a new cryptic protease. J Biol Chem.

[50] Liz MA, Fleming CE, Nunes AF, Almeida MR, Mar FM, Choe Y, Craik CS, Powers JC, Bogyo M, Sousa MM (2009). Substrate specificity of transthyretin: identification of natural substrates in the nervous system. Biochem J..

[51] Silva CS, Eira J, Ribeiro CA, Oliveira Â, Sousa MM, Cardoso I, Liz MA (2017). Transthyretin neuroprotection in Alzheimer’s disease is dependent on proteolysis. Neurobiol Aging.

[52] Alemi M, Gaiteiro C, Ribeiro CA, Santos LM, Gomes JR, Oliveira SM, Couraud PO, Weksler B, Romero I, Saraiva MJ, Cardoso I (2016). Transthyretin participates in beta-amyloid transport from the brain to the liver—involvement of the low-density lipoprotein receptor-related protein 1?. Sci Rep..

[53] Fleming CE, Saraiva MJ, Sousa MM (2007). Transthyretin enhances nerve regeneration. J Neurochem.

[54] Zawiślak A, Jakimowicz P, McCubrey JA, Rakus D (2017). Neuron-derived transthyretin modulates astrocytic glycolysis in hormone-independent manner. Oncotarget..

